# Running ‘LAPS’ Around nLD: Nuclear Lipid Droplet Form and Function

**DOI:** 10.3389/fcell.2022.837406

**Published:** 2022-02-01

**Authors:** Michael J. McPhee, Jayme Salsman, Jason Foster, Jordan Thompson, Sabateeshan Mathavarajah, Graham Dellaire, Neale D. Ridgway

**Affiliations:** ^1^ Department of Biochemistry & Molecular Biology, Dalhousie University, Halifax, NS, Canada; ^2^ Department of Pathology, Dalhousie University, Halifax, NS, Canada; ^3^ Department of Pediatrics, Dalhousie University, Halifax, NS, Canada

**Keywords:** nuclear lipid droplets, PML, lipin, CCTalpha, phosphatidylcholine, fatty acid

## Abstract

The nucleus harbours numerous protein subdomains and condensates that regulate chromatin organization, gene expression and genomic stress. A novel nuclear subdomain that is formed following exposure of cells to excess fatty acids is the nuclear lipid droplet (nLD), which is composed of a neutral lipid core surrounded by a phospholipid monolayer and associated regulatory and lipid biosynthetic enzymes. While structurally resembling cytoplasmic LDs, nLDs are formed by distinct but poorly understood mechanisms that involve the emergence of lipid droplets from the lumen of the nucleoplasmic reticulum and *de novo* lipid synthesis. Luminal lipid droplets that emerge into the nucleoplasm do so at regions of the inner nuclear membrane that become enriched in promyelocytic leukemia (PML) protein. The resulting nLDs that retain PML on their surface are termed lipid-associated PML structures (LAPS), and are distinct from canonical PML nuclear bodies (NB) as they lack key proteins and modifications associated with these NBs. PML is a key regulator of nuclear signaling events and PML NBs are sites of gene regulation and post-translational modification of transcription factors. Therefore, the subfraction of nLDs that form LAPS could regulate lipid stress responses through their recruitment and retention of the PML protein. Both nLDs and LAPS have lipid biosynthetic enzymes on their surface suggesting they are active sites for nuclear phospholipid and triacylglycerol synthesis as well as global lipid regulation. In this review we have summarized the current understanding of nLD and LAPS biogenesis in different cell types, their structure and composition relative to other PML-associated cellular structures, and their role in coordinating a nuclear response to cellular overload of fatty acids.

## Introduction

The lipid droplet (LD) is a unique cellular organelle composed of a surface monolayer of phospholipids and proteins surrounding a neutral lipid core containing triacylglycerides (TAG^1^) (Walther et al., 2017), steryl esters (Shen et al., 2016) and/or retinyl esters (Orban et al., 2011). Nutrient stress or excess fatty acids promote the storage of neutral lipids in LDs, which can be subsequently released by ester hydrolases to provide energy, lipid precursors for membrane biogenesis and signalling molecules (Henne et al., 2018). LDs therefore sequester essential biomaterials, while protecting the cell from the lipotoxic effects of excess fatty acids and cholesterol that can promote ER stress and mitochondrial dysfunction, which promote cell death (Olzmann and Carvalho, 2019). The defective storage of lipids in LDs has profound pathophysiological consequences. In the case of TAG, unilocular LDs in adipocytes are the primary storage depot but hepatocytes and other cells are also capable of short-term storage and release of fatty acids from LDs. However, chronic exposure of hepatocytes to fatty acids causes non-alcoholic fatty liver disease (NAFLD) (Diehl and Day, 2017), a common form of hepatic steatosis caused by fatty acid-induced ER and mitochondrial stress (Cazanave et al., 2010; Mantzaris et al., 2011), defective lipophagy of LDs (Singh et al., 2009) and lipid activation of pro-apoptotic transcriptional pathways (Barreyro et al., 2007). When combined with an inflammatory insult, NAFLD can progress to non-alcoholic steatohepatitis, which is associated with hepatic fibrosis and cirrhosis. NAFLD is a major risk factor for chronic liver disease and contributes to rising rates of liver transplantation in developed countries (Pais et al., 2016).

The lipid storage function of LDs relies on a complement of core proteins on their surface that regulate the storage and release of lipids in response to nutrient signaling. Proteomic analysis has revealed an expanded repertoire of associated proteins that suggest a wide-ranging role for LDs in cell physiology. These include the MAX dimerization (MLX) protein and related glucose-sensing transcription factors (Mejhert et al., 2020), histone storage (Cermelli et al., 2006), nuclear pore protein expression (Kumanski et al., 2021), clearance of misfolded and ubiquitinated proteins (Ohsaki et al., 2008; Moldavski et al., 2015) and immune responses to viral and bacterial infection (Bosch et al., 2021). Consequently, our understanding of LD biology has evolved from that of lipid storage depots to one of dynamic organelles that functionally intersect with many cellular metabolic and signalling activities. This has been further challenged by the discovery and characterization of nuclear lipid droplets (nLD) that share features with their cytosolic counterparts but are unique in terms of biogenesis, their associated proteins and lipids, and ultimately their cellular activities (Sołtysik et al., 2019).

nLDs were first characterized in normal and transformed hepatocytes (Layerenza et al., 2013; Uzbekov and Roingeard, 2013; Wang et al., 2013) and later Caco2 intestinal epithelial cells (Yue et al., 2020), reflecting their biogenesis from lipoprotein precursors (Soltysik et al., 2019). nLDs are rarely observed in common laboratory cell lines and tissues that do not secrete lipoproteins. An exception is U2OS osteosarcoma cells that contain abundant nLDs when incubated with oleate (Ohsaki et al., 2016). nLDs have been identified in yeast under nutrient stress conditions and certain mutational backgrounds (Romanauska and Kohler, 2018) and intestinal and germ cells of *Caenorhabditis elegans* (Mosquera et al., 2021). As will be discussed, the apparent restricted distribution of nLDs reflects both their unique biosynthetic origins and limited investigation in other cells and organisms.

Since cytoplasmic lipid droplets (cLD) are more abundant than their nuclear counterparts, nLDs visualized by wide-field microscopy could be cLDs trapped in invaginations of the nuclear envelope (NE). However, serial sections of human liver revealed that nLDs are not connected to the NE and associate with heterochromatin (Uzbekov and Roingeard, 2013). nLDs visualized by confocal microscopy of rat hepatocytes were not associated with nuclear lamina, and could be isolated from purified nuclei (Layerenza et al., 2013). A detailed lipidomic analysis of isolated nLDs is not available; however, lipid class analysis indicated they contain more cholesterol ester, cholesterol and phospholipids, less TAG, and a higher protein-to-lipid ratio relative to cLDs (Layerenza et al., 2013). These features reflect the smaller size and larger surface-to-volume ratio of nLDs. A proteomic analysis of purified nLDs from rat liver revealed a variety of cytoskeletal proteins, transcription and translation factors, histones and carboxyl esterase 1 (Lagrutta et al., 2021). Interestingly, the proteome of purified nLDs did not contain any nLD-associated proteins identified by microscopy-based methods (Ohsaki et al., 2016; Soltysik et al., 2019; Lee et al., 2020; Soltysik et al., 2021). These differences likely reflect the conditions used to isolate the nLDs, which could strip loosely associated proteins. As a result, to more fully assess the nLD proteome, gentler *in situ* methods are required that do not involve cellular disruption and biochemical purification, such as proximity labelling and mass spectrometry of *in vivo* labelled protein complexes. In the following sections, we will highlight our current understanding of the proteins and lipids involved in the biogenesis of nLDs at the inner nuclear membrane (INM) and potential functions in the nucleoplasm, with a focus on the association of nLDs with promyelocytic leukemia (PML) protein in lipid-associated PML structures (LAPS).

## Mechanisms of Nuclear Lipid Droplet Biogenesis

### Nuclear Lipid Droplet Biogenesis on the Inner Nuclear Membrane

**FIGURE 1:**
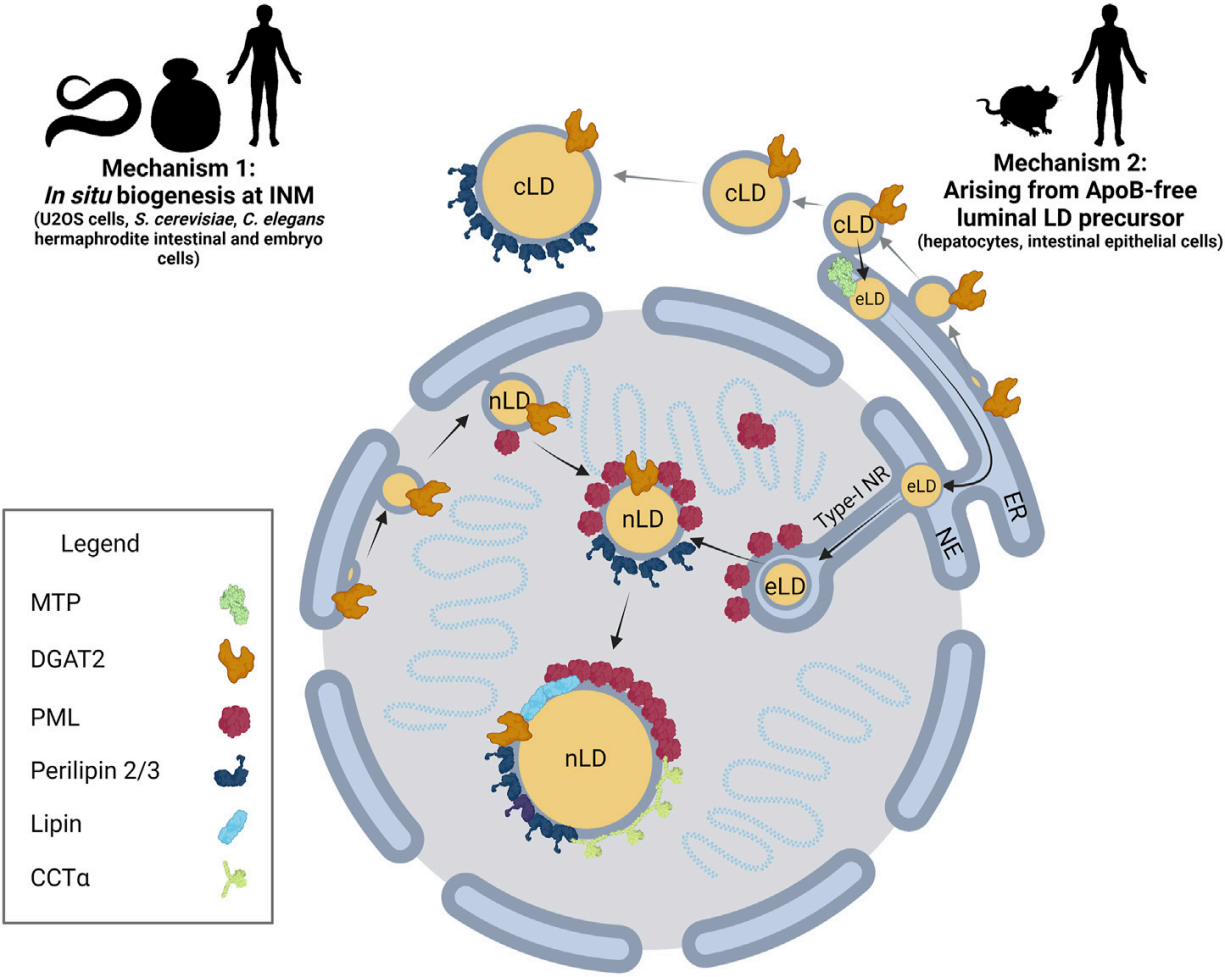
nLD biogenesis in mammalian cells. Two mechanisms have been identified for nLD formation. Mechanism 1: Much like cLD biogenesis, nLD biogenesis in U2OS cells, *S. cerevisiae*, and *C. elegans* involves *in situ* TAG synthesis at the INM facilitated by lipid biosynthetic enzymes. nLD biogenesis is seipin-dependent in *S. cerevisiae*, whereas the process is seipin-independent in U2OS cells. Mechanism 2: In specialized lipoprotein-exporting mammalian cells like hepatocytes and intestinal epithelial cells, ApoB-free eLDs form in an MTP-dependent manner and subsequently migrate through the lumen of the ER into the lumen of the NE. Next, eLDs enter into type-I NR invaginations of the INM that extend into the nucleoplasm. PML-II localizes to INM at lamin-deficient regions, possibly facilitating translocation of the LD through ruptures of the INM into the nucleoplasm. In mammalian cells more generally, lipid biosynthetic enzymes DGAT2, Lipin-1 and CCTα**,** LD coat protein perilipin-3, and PML are all present at the surface of nLDs. The binding of lipid biosynthetic enzymes and the formation of LAPS are two commonalities of nLDs irrespective of their biogenesis in mammalian cells, suggesting a possible conserved function for these structures.

The immiscibility of hydrophobic neutral lipids in the cytosol is ultimately the key physical property driving nLD and cLD formation, growth and stability. Eukaryotic cells coordinate each of these processes through a dynamic, non-stochastic, and tightly regulated mechanism, allowing cells to effectively respond to changes in energy status, substrate availability and cellular stress. nLD biogenesis occurs by at least two known pathways: 1) *in situ* biogenesis at the INM and nLD budding into the nucleoplasm and 2) ER luminal lipid droplets (eLD) that migrate into type-1 nucleoplasmic reticulum (NR) invaginations that rupture to release a nascent nLDs into the nucleoplasm (Figure 1).

**FIGURE 2:**
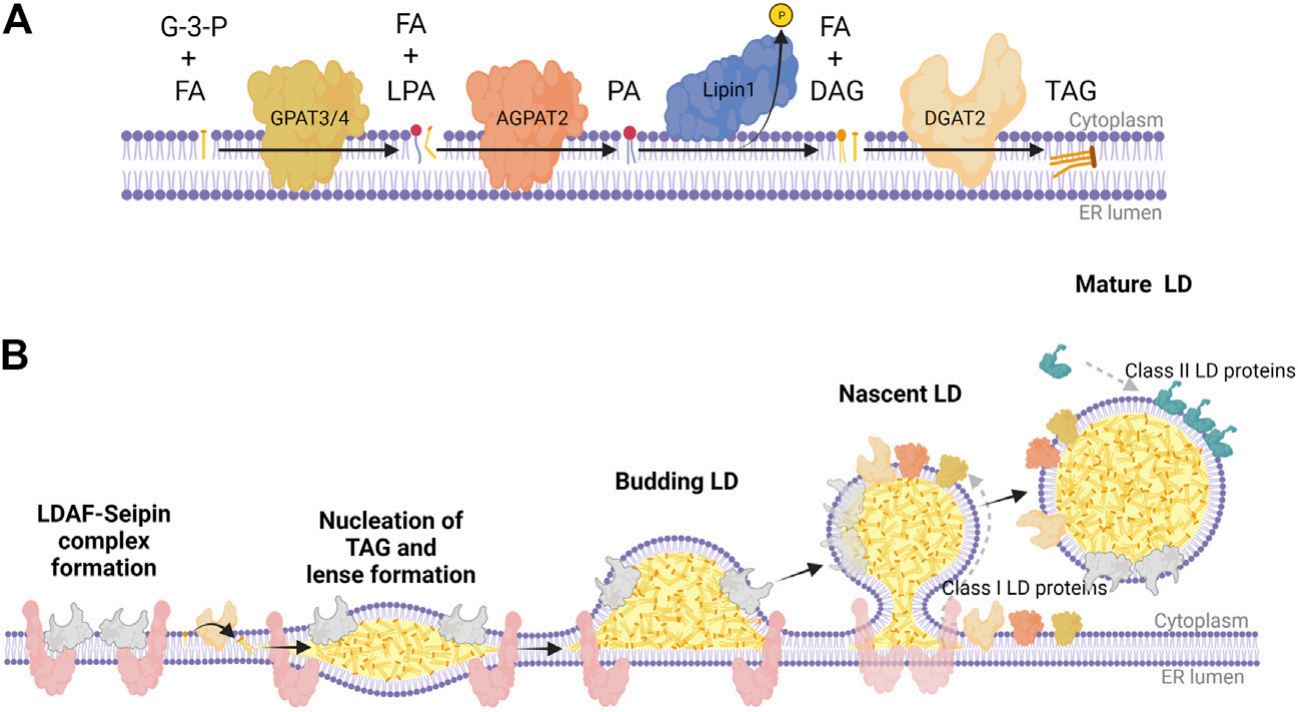
The Kennedy pathway for TAG synthesis and LD biogenesis in mammalian cells. **(A)** Biosynthetic enzymes of the Kennedy pathway act sequentially to synthesize triacylglycerol (TAG) at the ER membrane; GPAT3/4 synthesizes lysophosphatidic acid (LPA) from glycerol 3-phosphate (G-3-P) and fatty acids (FA), AGPAT2 synthesizes phosphatidic acid (PA) from LPA and FA, Lipin-1 hydrolyzes PA to diacylglycerol (DAG), and DGAT2 catalyzes the final acylation to form TAG. **(B)** TAG nucleates between the two leaflets of the ER membrane bilayer, which is partly facilitated by a complex of LDAF and seipin at distinct domains throughout the ER. These points of TAG nucleation develop into lens-like structures that proceed to bud into the cytoplasm as a budding LD coated with LDAF as it dissociates from seipin. As seipin funnels TAG and DAG into nascent LDs, lipid biosynthetic enzymes (class I LD proteins) like GPAT3/4, AGPAT2, and DGAT2 transfer across membrane bridges to the surface monolayer, further facilitating the maturation of LDs. Once the mature LD separates from the ER, it recruits class II LD proteins like perilipin-2/3, which coat the surface to regulate access of LDs to lipases and autophagy proteins. This graphic was created with Biorender.com.

*In situ* nLD biogenesis is documented in *Saccharomyces cerevisiae* (Romanauska and Kohler, 2018) as well as U2OS cells (Lee et al., 2020; Soltysik et al., 2021). While nLDs are particularly enriched in cholesterol esters (Layerenza et al., 2013), nLD biogenesis in most studies in mammalian cells is induced with exogenous oleate. The mechanism for formation of TAG-enriched nLDs appears to have many features in common with that for cLD formation in the ER (Thiam et al., 2013; Walther et al., 2017) (Figure 2). TAG is synthesized *de novo* from glycerol-3-phosphate and fatty acids by the concerted activity of glycerol 3-phosphate acyltransferases (GPAT) and 1-acylglycerol 3-phosphate acyltransferases (AGPAT) to produce phosphatidic acid (PA), which is dephosphorylated by Pah1 (*S. cerevisiae)* and Lipins (mammals) to produce diacylglycerol (DAG). Finally, DAG acyltransferases (DGAT) 1 and DGAT2 produce TAG (Figure 2A). These TAG lipid synthetic enzymes concentrate at specific sites of the endoplasmic reticulum (ER) membrane to facilitate the nucleation of *de novo* synthesized neutral lipid between leaflets of phospholipid bilayer (Figure 2B). These lens-like structures accumulate lipids and bud into the cytoplasm to produce nascent cytoplasmic LDs (cLD). cLDs continue to mature or ‘ripen’ through three mechanisms: 1) seipin-dependent membrane bridges that connect the cLD monolayer with the cytosolic leaflet of the ER, allowing the diffusion of proteins and lipids to the LD (Wang et al., 2016; Salo et al., 2019), 2) targeting of GPAT4, AGPAT4, and DGAT2 to the LD to facilitate *in situ* TAG synthesis (Wilfling et al., 2013; McFie et al., 2018; Olarte et al., 2022) and 3) coalescence of lipid droplets (Thiam et al., 2013).

In the case of oleate-treated U2OS cells, nLD biogenesis involves an *in situ* pathway. Similar to cLDs (Figure 2), AGPAT2, GPAT3/4, Lipin-1 and DGAT1/2 are localized to the INM of U2OS cells and required for nLD biogenesis (Lee et al., 2020; Soltysik et al., 2021). Acyl-CoA synthetase long chain family member 3 (ASCL3), lyso-phosphatidylcholine acyltransferase 1 (LPCAT1), GPAT3/4, Lipin-1β, and DGAT2 also localize to the surface of the nLD and could provide TAG and phosphatidylcholine (PC) for growth of the nLD after it buds into the nucleoplasm (Soltysik et al., 2021). mTORC1 inhibition promotes the nuclear translocation of Lipin-1β and increased nLDs (Soltysik et al., 2021). Lipin-1α and -1β are enriched on the surface of a significant fraction of nLDs containing DAG suggesting they provide substrate for DGAT and TAG biosynthesis (Lee et al., 2020). nLDs in oleate-treated U2OS also serve as a platform for recruitment and activation of CTP:phosphocholine cytidylyltransferase α (CCTα), the rate-limiting enzyme in the CDP-choline pathway for PC synthesis (Figure 1) (Lee et al., 2020). Similar to the activation of CCT1 on cLDs in *Drosophila melanogaster* S2 cells (Krahmer et al., 2011), CCTα activation on nLDs increases cellular PC synthesis to expand the TAG storage capacity in cLDs, or to provide PC to reduce fatty acid-induced ER stress (Soltysik et al., 2019).

In contrast to cLD biogenesis in the ER (Figure 2B), the formation of nLDs in U2OS cells is seipin-independent (Soltysik et al., 2021). Seipin has a well-recognized role in the nucleation of cLD formation in the ER where it forms an oligomeric ring complex with lipid droplet assembly factor 1 (LDAF1) that acts to concentrate DAG and TAG, and facilitate protein and lipid transfer from the ER to nascent cLDs (Figure 2B) (Wang et al., 2016; Yan et al., 2018; Chung et al., 2019; Salo et al., 2019). Seipin also interacts with GPAT3/4, AGPAT2, and Lipin1 to concentrate the production of TAG at specific sites in the ER (Sim et al., 2012; Sim et al., 2020). Interestingly, RNAi-mediated seipin knockdown in U2OS cells promoted increased nLD formation by an ACSL3 and Lipin-1β-dependent mechanism (Soltysik et al., 2021). This correlated with reduced expression of the splicing factor TRA2B leading to increased expression and translocation of Lipin-1β into the nucleus. The mechanism for seipin induction of TRA2B is not clear but the resultant increase in Lipin-1β activity provides an explanation for the observed increase in nLDs. Since seipin establishes the site of cLD biogenesis and membrane tethers required for expansion, other factors at the INM must be responsible for nucleating and tethering nLDs in U2OS cells. While there is evidence for nLD biogenesis in the INM of U2OS cells, it is uncertain whether this pathway is responsible for formation of nLDs in other mammalian cells or works in conjunction with the eLD pathway for nLD formation in hepatocytes (see below).

nLDs form *in situ* at the INM of *S. cerevisiae* by a seipin-dependent mechanism that bares many of the features of cLD biogenesis (Romanauska and Kohler, 2018). Increased availability of PA resulting from genetic mutations of lipid biosynthetic enzymes facilitates DAG formation and nLD budding at the INM (Romanauska and Kohler, 2018). For example, a temperature-sensitive CDP-diacylglycerol synthase 1 (*Cds1*) mutant grown at the non-permissive temperature had a 6-fold increase in nLDs due to the shift toward TAG storage. Cds1 synthesizes CDP-DAG, the common substrate for synthesis of *S. cerevisiae* phospholipids, and is one of many lipogenic enzymes upregulated by the Ino2-Ino4 transcription factors (Carman and Han, 2011). *INO2-INO4* deletion or antagonism by its repressor Opi1 recapitulated a similar nLD phenotype as *Cds1* mutants (Romanauska and Kohler, 2018). Key enzymes in yeast TAG synthesis, Pah1, Dgk1 and Cds1, were localized to the INM, as were the substrates for TAG synthesis, PA and DAG. The TAG lipase Tgl5 was found on the surface of nLDs providing a plausible mechanism for nLD turnover. A recent study addressed how the lipid synthesis and storage capacity of the INM maintains an optimal lipid environment by overcoming unsaturated fatty acid-induced stress (Romanauska and Kohler, 2021). Membrane fluidity biosensors were used to identify a Mga1-Ole1 transcriptional circuit for unsaturated fatty acid synthesis that induced storage in cLDs and suppressed nLD formation by reducing seipin and PA levels in the INM. This mechanism for unsaturated fatty acid detoxification would maintain the optimal phospholipid fluidity and packing in the INM.

Unlike mammalian cells, seipin is required for nLD formation in *S. cerevisiae* (Romanauska and Kohler, 2018). Seipin was found to localize to the INM using bimolecular fluorescence complementation with the nuclear pore protein Nup60, and ultrastructural analysis confirmed the presence of seipin-dependent bridges between the outer leaflet of the INM and the nLD monolayer. nLD biogenesis occurs in seipin-deficient *S. cerevisiae* but membrane bridges are notably absent and nLDs are mostly embedded within inclusions within the INM.

Some clues as to the evolutionary emergence of nLDs in eukaryotes are provided by comparing yeast and amoeba. *Dictyostelium discoideum* expresses a seipin homologue but nLDs have not been observed (Du et al., 2013). The localization of *D. discoideum* seipin was restricted to a subset of foci and rings co-localizing to the ER and cLDs at the edge of the plasma membrane (Kornke and Maniak, 2017). While the loss of seipin in amoebae resulted in fewer but larger cLDs, there was no change in overall TAG production (Kornke and Maniak, 2017). The lack of nuclear membrane localization of *D. discoideum* seipin may explain why the species lacks nLDs. A transition in seipin localization from the ER to the ER-INM during opisthokont evolution could contribute to the formation of nLDs that are observed in yeast.

Different classes of nLDs were identified in hermaphrodite intestinal and germ cell nuclei of *C. elegans* based on association with chromatin and the lamina (Mosquera et al., 2021). The frequency of nLDs in the nuclei of intestinal cells ranged from 5 to 20% and increased with developmental stage. Cells usually contained a single nLD that could, on occasion, occupy one-third the nuclear volume. Transmission electron microscopy imaging of intestinal nuclei revealed nLDs that were; 1) free within the nucleoplasm, 2) between the peripheral heterochromatin and nuclear lamina, 3) coated by heterochromatin, lamina and membrane, and 4) surrounded by double membrane due to in-pocketing in type-1 NR (Mosquera et al., 2021). Gonadal germ cells acquire lipid from intestinal lipoproteins for storage in cLDs during development, and a small population of these cells (<20%) also produced nLDs. Unlike intestinal cells, nLDs in germ cells did not have associated lamina, heterochromatin or surrounding membrane, and were associated with rapid oogenesis. While the function(s) of nLDs in *C. elegans* is unclear, it was observed that nLDs were frequently associated with sites of nuclear rupture and repair in intestinal cells, suggesting they may interfere with chromatin organization, lamina integrity and/or repair pathways. In contrast, nLDs were not associated with nuclear damage or cell survival in germ cells. Aside from a single report showing association of the hydroxysteroid reductase DHS-9 with intestinal nLDs by immunofluorescence microscopy (Liu et al., 2018), the proteome of *C. elegans* nLDs is unknown.

The close proximity of intestinal nLDs with the INM and type-I NR suggests they could originate by an *in situ* mechanism similar to that proposed for U2OS cells and yeast (Figure 1). However, intestinal cells of *C. elegans* produce a lipoprotein-like paralog of ApoB called vitellogenin, a yolk protein exported from intestinal cells into the coelom where it is taken up by the hermaphrodite gonads to support embryogenesis and fertility (Perez and Lehner, 2019). *C. elegans* also expresses an orthologue of MTP (Dsc4) that is targeted to the ER and supports lipoprotein secretion (Rava and Hussain, 2007). These findings indicate that nLD biogenesis could involve a TAG-rich lipoprotein precursor, as reported in hepatocytes (see below) (Soltysik et al., 2019).

In an effort to determine if *C. elegans* nLDs affect germ cell viability, a mutational screen was used to identify genes that regulated nLD abundance and size (Mosquera et al., 2021). Mutants of SEIP-1, NEMP-1 and the COPII coat proteins COPA-1 and COPB-2 increased nLD size and number in germ cells but did not affect viability. SEIP-1 encodes the *C. elegans* homologue of seipin, the absence of which caused the appearance of large nLDs in germ cells. This is similar to the effect of seipin knockdown in U2OS cells (Soltysik et al., 2021) and suggests an indirect role for SEIP-1 in nLD biogenesis in the cytoplasm. NEMP-1 is a poorly characterized integral membrane protein that localizes to the nuclear lamina and contains a RanGTP binding domain (Shibano et al., 2015). The loss of COPA-1 and COPB-2 coat proteins could increase nLD formation indirectly by promoting unfolded protein stress in the ER or by the delivery of enzymes that control TAG storage, as proposed for COPII vesicle transport of adipose triglyceride lipase and perilipin-2 to cLDs in mammalian cells (Soni et al., 2009).

Finally, it should be noted that neither the yeast or *C. elegans* genomes encode a PML ortholog. As such, these data indicate that nLD formation in these species does not strictly require PML, as is the case for mammalian cells (see below). However, it remains to be determined if a paralogous PML-like protein, for example containing the highly conserved tripartite motif (TRIM) domain found in PML and many E3 ubiquitin ligases (Gushchina et al., 2018), exists in these species in association with nLDs.

### Nuclear Lipid Droplet Biogenesis From ER Luminal Lipid Droplet

Another pathway for nLD formation occurs in cells that secrete TAG-rich lipoproteins, such as hepatocytes (Ohsaki et al., 2016; Soltysik et al., 2019) and intestinal epithelial Caco2 cells (Yue et al., 2020) (Figure 1). Hepatocytes assemble TAG-rich ApoB-containing very low-density lipoproteins (VLDL) in the ER lumen from which they are exported to the *cis-*Golgi via COPII transport vesicles for eventual secretion into circulation. Microsomal triglyceride transfer protein (MTP) in the ER lumen transfers TAG and phospholipids to newly synthesized ApoB to form a VLDL precursor (Lehner et al., 2012). MTP also transfers lipids to eLDs that are ApoB-deficient and fuse with ApoB-containing precursors to produce VLDL. Under conditions of ER stress and increased TAG synthesis, a fraction of these eLDs traffic from the ER lumen into type-I NR invaginations of the INM (Soltysik et al., 2019) (Figure 1). eLDs containing ApoE and ApoCIII translocate into the nucleoplasm through regions of the inner leaflet of the INM enriched in PML-II but depleted of lamin A, lamin B receptor and SUN1/2 (Ohsaki et al., 2016; Soltysik et al., 2019). PML-II is the only isoform involved in eLD egress into the nucleoplasm. Type-I NR seems to be dispensable for nLD formation since stimulation of NR formation by tunicamycin had no effect (Soltysik et al., 2019). Once in the nucleoplasm, nLDs can expand or mature by recruitment DGAT2 and CCTα to increase *de novo* synthesis of TAG and PC synthesis, respectively (Ohsaki et al., 2016; Soltysik et al., 2019).

## Functions of Nuclear Lipid Droplets and Lipid-Associated Promyelocytic Leukemia Structures

### Platforms for the Regulation of Lipid Synthesis

nLDs represent <10% of the LD pool (Ohsaki et al., 2016; Lee et al., 2020), are TAG-poor relative to cLDs (Layerenza et al., 2013) and lack proximity to the mitochondria and ER, the primary loci of lipid oxidation and synthesis, respectively. Thus nLDs are unlikely to serve as energy storage reservoirs but could have additional functions related to cell signalling, protein storage and mitigation of ER stress. nLDs in hepatocytes and intestinal epithelial cells have an origin and functions that are linked to the absorption, repackaging and secretion of lipids in lipoproteins (Soltysik et al., 2019). During ER stress, ApoB is degraded co-translationally leading to accumulation of eLDs and release into the nucleoplasm through type-1 NR invaginations to form nLDs. The subsequent recruitment and activation of CCTα on nLDs increases PC synthesis, which is negatively regulated by displacement from nLDs by perilipin-3. Increased PC synthesis could mitigate ER stress by; 1) expanding the ER network to accommodate unfolded proteins and 2) providing surface monolayer phospholipids for the packaging of fatty acids into TAG for storage in cLDs and secretion in VLDL. It is notable that nLD formation did not occur in hepatocytes treated with tunacimycin alone; however, the unfolded protein response induced by tunicamycin enhanced nLD formation in response to oleate (Soltysik et al., 2019).

It is currently unknown why CCTα translocates to the surface of nLDs rather than the INM, which is commonly observed in oleate-treated cells that lack nLDs (Wang et al., 1995; Lagace and Ridgway, 2005; Gehrig et al., 2008) and during 3T3-L1 preadipocyte differentiation (Aitchison et al., 2015). The association of the domain M α-helix of CCTα with membranes is enhanced by low PC content and the presence of lipid activators, such as DAG, PA and fatty acids (Cornell and Ridgway, 2015). LAPS appear to be a preferred substrate for CCTα translocation as PML-knockout U2OS cells had a partial shift of CCTα to the NE (Lee et al., 2020). The preferred association of CCTα with nLDs could be driven by a unique protein and/or lipid composition. However, the eLD precursors of nLDs and LAPS have a similar lipid composition as cLDs (Wang et al., 2007), and the composition of hepatic nLDs does not indicate enrichment in CCTα activating lipids (Layerenza et al., 2013). Interestingly, LAPS are enriched in DAG, a known activator of CCTα; however, the DAG content of LAPS did not correlate with enrichment in CCTα (Lee et al., 2020). PA was only observed on nuclear puncta and infrequently on small nLD in U2OS cells and is also unlikely to be a factor in CCTα activation (Soltysik et al., 2021).

Nuclear CCTα controls the rate of PC synthesis by supplying CDP-choline to choline/ethanolamine phosphotransferase (CEPT) and choline phosphotransferase (CPT) in the ER and ER/Golgi, respectively (Henneberry et al., 2002). This cellular topology implies that CDP-choline synthesized by CCTα is transported to the cytoplasm for PC synthesis. However, a split-GFP reporter screen identified the yeast homologues of CPT and CEPT, Cpt1p and Ept1p, in the INM (Smoyer et al., 2016). In support of INM localization of CEPT, approximately 10% of the epitope-tagged enzyme was in the NE of CHO cells (Gehrig and Ridgway, 2011) and deuterated choline-labelled lipids were detected in the NE using nanoscale-secondary ion mass spectrometry (Drozdz et al., 2017). The last two studies do not preclude the possibility that CEPT is active on the outer nuclear membrane and newly synthesized PC undergoes lateral diffusion to the INM at nuclear pores (Barger et al., 2022).

### Regulation of Chromatin Structure, Gene Expression and Cell Signalling

There is indirect evidence that nLDs could perturb chromatin structure and gene expression. nLD formation from eLDs occurs at sites of lamin depletion in the INM (Ohsaki et al., 2016), which could affect the interaction and organization of chromatin (Dechat et al., 2008). nLDs isolated from rat liver have associated histones, including variants of H2A, H2B, H3.3 and H4 (Lagrutta et al., 2021). Similarly, cLDs in a variety of eukaryotic species are high capacity storage sites for histones (Welte, 2007), both buffering the genotoxic effects of histone excess and increasing supply to match DNA replication, as demonstrated during *Drosophila* embryogenesis (Li et al., 2012). While the role of nLDs in histone regulation is less clear, the PML NB-associated protein death domain-associated protein 6 (DAXX) is a H3.3 chaperone (Drane et al., 2010), and H3.3 localization to PML-NB regulates its association with chromatin (Corpet et al., 2014). Whether this association with H3.3 also occurs on LAPS is unknown but supports the notion that nLDs and LAPS could modify chromatin and gene expression (next section). Studies in *C. elegans* have shown direct interaction between nLD and chromatin; however, it was not determined whether these interactions are facilitated by proteins or lipids, and if gene expression was altered (Mosquera et al., 2021). While not fully investigated, understanding the interplay between nLDs and LAPS with chromatin appears to be a promising way to better understand nLD function.

nLDs could also influence gene expression by providing the ligands for the activation of nuclear transcription factors. For example, nLDs could serve as a source of cholesterol, fatty acids and PC ligands and precursors for liver X receptor (Janowski et al., 1996), peroxisome proliferator-activated receptors (PPAR) (Tanaka et al., 2017) and LRH-1 (Musille et al., 2012). More directly, the recruitment of the transcriptional co-activator Lipin-1 to nLDs in U2OS and HuH7 cells (Lee et al., 2020; Soltysik et al., 2021) could affect the expression of multiple genes involved in fatty acid catabolism and storage. Lipin-1 is a co-activator of PPARγ, which is also activated by fatty acid ligands (Tanaka et al., 2017). Mechanistically, this involves Lipin-1 activation of PPARγ co-activator 1A (PGC1A) to promote the transcription of fatty acid oxidation genes (Finck et al., 2006). Lipin-1 also influences expression of lipid catabolic genes through its direct interaction with hepatic nuclear factor 4α (Chen et al., 2012). Nuclear Lipin-1 is also a repressor of the lipogenic transcription factor sterol regulatory element binding protein (SREBP) 1c by a mechanism involving mTORC1 phosphorylation (Peterson et al., 2011). Lipin-1 phosphorylation by mTORC1 causes its retention in the cytoplasm, while dephosphorylation by the NE complex of C-terminal domain nuclear envelope protein (CTDNEP) and nuclear envelope protein 1 regulatory subunit 1 (NEP1R1) leads to nuclear import (Csaki et al., 2013). Treatment of U2OS cells with an mTORC1 inhibitor and oleate increased nLD formation, which was dependent on the catalytic and transcriptional co-activation activity of Lipin-1 (Soltysik et al., 2021). The AAA+ ATPase Torsin A is another important regulator of lipid metabolism via its inhibitory effects on Lipin activity in the nucleus (Grillet et al., 2016; Jacquemyn et al., 2021). In *Drosophila*, Torsin was shown to promote dissociation of the NEP1R1-CTDNEP1 phosphatase complex from the NE, resulting in Lipin exclusion from the nucleus (Jacquemyn et al., 2021). Torsin dysregulation was associated with nuclear pore assembly defects in *Drosophila* fat body cells. However, mice with an hepatic knockout of lamina-associated polypeptide 1 (LAP1), which is required for Torsin A activity, displayed impaired VLDL secretion, hepatic steatosis and nLD accumulation (Shin et al., 2019). This increased nLD phenotype could result from loss of negative regulation of Lipin-1 via Torsin A/NEP1R1-CTDNEP1 and increase eLD translocation into the nucleus due to inhibition of VLDL assembly. By virtue of its role as a key regulator of fatty acid incorporation into TAG, Lipin-1 has the potential to connect lipid metabolism on nLD to the transcriptional activation of pathways that control fatty acid homeostasis.

## Structure and Function of Promyelocytic Leukemia-Associated Sub-cellular Domains and Lipid-Associated PML Structures

### The Structure and Function of Classical Promyelocytic Leukemia Nuclear Bodies

**FIGURE 3:**
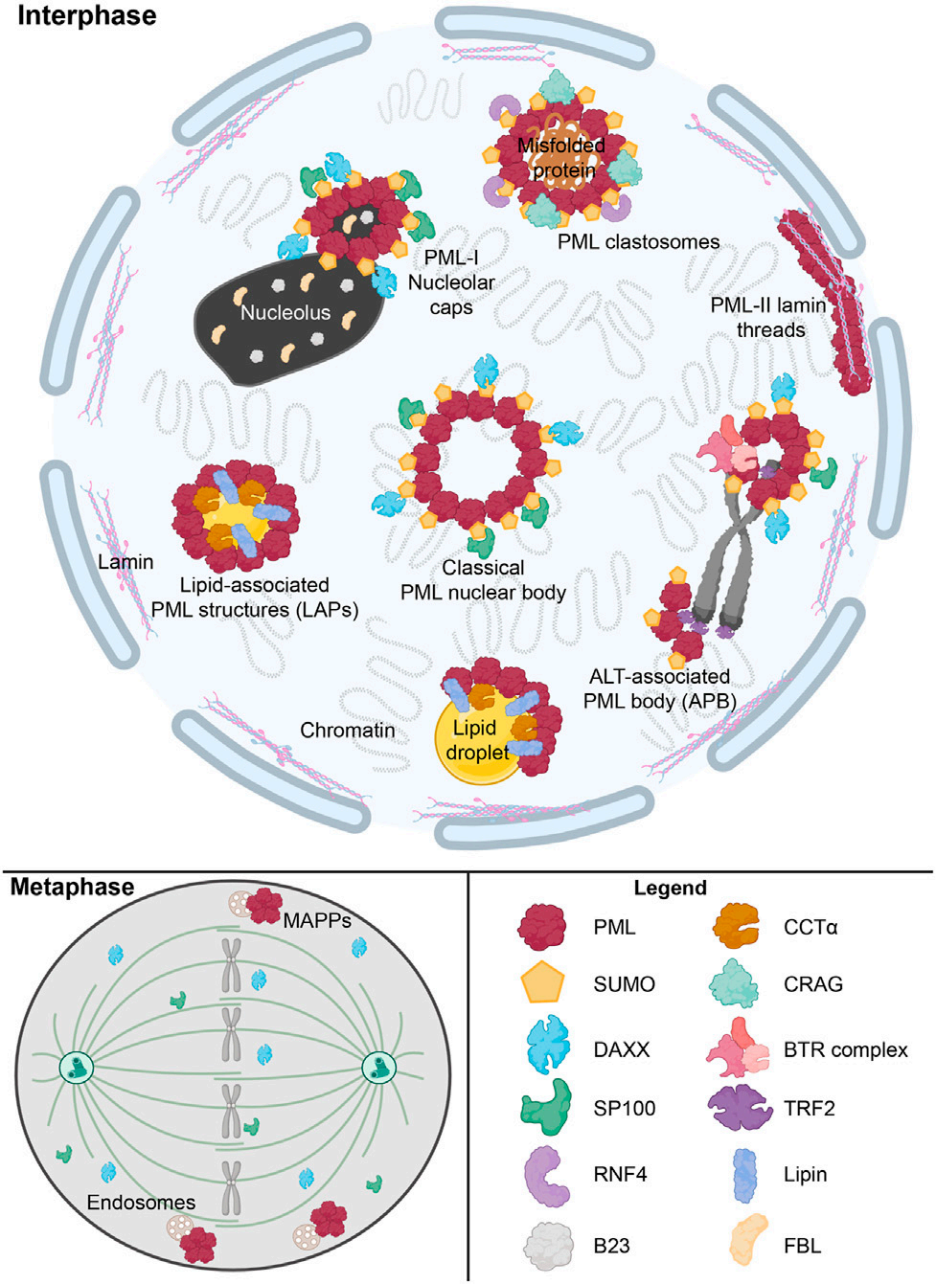
Overview of nuclear PML structures and their interactors. The formation of canonical phase-separated spherical PML NB is driven by protein oligomerization and SUMO-SIM interactions, which also recruit other proteins such as DAXX and SP100. However, other novel PML structures form under specific stimuli. These include ALT-associated PML bodies (APB), PML-I nucleolar caps and PML clastosomes. SUMO-independent LAPS form on nLDs and host lipid biosynthetic enzymes such as Lipin1 and CCTα. During mitosis, SUMO-independent structure known as mitotic accumulations of PML protein (MAPP) form and are tethered to endosomes. In certain disease states such as with Hutchinson-Gilford progeria syndrome, PML-II lamin threads are formed. These aforementioned PML structures uniquely interact with a number of proteins, such as RNF4, CRAG, TRF2, and the BTR complex, to modulate normal cellular functions and the cell’s response to stress states. This graphic was. created with Biorender.com.

As mentioned in previous sections, a defining feature of nLDs in mammalian cells is their association with PML to form lipid-associated PML structures called LAPS, one of numerous nuclear substructures containing PML that are summarized in Table 1 and Figure 3. The PML gene contains 9 exons which are subjected to alternative mRNA splicing, resulting in at least 6 nuclear isoforms containing a common N-terminal RING-B-box-coiled-coiled (RBCC) motif (also referred to as the TRIM domain) and variable C-terminal tails (Jensen et al., 2001; Nisole et al., 2013; Gushchina et al., 2018) Collectively, the PML isoforms form the molecular basis for the formation of PML NBs (Figure 3). These PML NBs are dynamic, heterogeneous sub-nuclear structures that serve as regulatory hubs for over 150 associated nuclear proteins, consisting primarily of transcription factors and chromatin remodeling protein (Van Damme et al., 2010). Thus PML NBs are implicated in a variety of key cell survival pathways, including the DNA damage response, senescence, gene expression regulation, apoptosis, nuclear proteolysis and the antiviral response (Pearson and Pelicci, 2001; Bischof et al., 2002; Dellaire and Bazett-Jones, 2004; Dellaire et al., 2006a; Villagra et al., 2006; Bernardi et al., 2008; Attwood et al., 2020). In addition, many components of the small-ubiquitin like modifier (SUMO) machinery, such as SUMO proteases (i.e., SENPs) and SUMO ligases (i.e., UBC9, PIAS), associate with PML NBs making these structures hubs for SUMO biology within the cell (Van Damme et al., 2010; Hattersley et al., 2011; Sahin et al., 2014; Brown et al., 2016; Barroso-Gomila et al., 2021).

**TABLE 1:** Nuclear structures containing PML.

PML structure	Description	Conditions/Stimuli	Localization	Key components	Diagnostic criteria	References
PML NB	PML nuclear bodies	Normal cells	4–30 bodies per cell	SUMO, SP100, DAXX	SUMO, SP100, DAXX	Song et al. (2004), Cheng and Kao (2012), Banani et al. (2016), Hoischen et al. (2018), Corpet et al. (2020)
LAPS	Lipid-associated PML structures	Excess oleate	nLD	CCTα, Lipin1, DAG	Visualized with lipid dyes	Ohsaki et al. (2016), Lee et al. (2020)
APB	ALT-associated PML bodies	ALT-positive cancer cells	Telomere- associated PML bodies	SUMO, SP100, DAXX, BTR complex, TRF2, telomeric DNA	Co-localization withTRF2 in *tert-negative* cells	Chung et al. (2012), Loe et al. (2020)
MAPP	Mitotic accumulation of PML proteinsl	Mitosis	Endosome- associated	PML protein aggregates	Co-localization with EEA1, TfR	Dellaire et al. (2006b); Palibrk et al. (2014)
PML patches	Nuclear lamin- associated patches/threads	Hutchinson-Gilford progeria cells; senescent cells	PML-II on nuclear lamina, type-1 NR	SUMO	Reduced associated with DNA repair proteins (yH2AX, RPA32, MRE11)	Wang et al. (2020)
				DAXX		
				Progerin		
Nucleolar caps	Senescence-associated PML-I caps	Senescent cells	surrounding nucleolar fragments and blebs	SUMO, DAXX, SP100,B23, DHX9,FBL	Co-localization with nucleolus-fibrillar center	Condemine et al. (2007), Imrichova et al. (2019)
PML clastosomes	PolyQ-associated PML rings at nuclear protein inclusions	Misfolded polyQ proteins, UV	Enlarged nuclear ring	CRAG	Co-localization with ubiqutitin-positive inclusions; SUMO	Qin et al. (2006), Guo et al. (2014), Janer et al. (2006)
				RNF4		
				SUMO		
				PML		

PML NBs range in diameter from 0.1 to 1 μm, with mammalian cells hosting 4–30 bodies (Table 1) (Banani et al., 2016; Hoischen et al., 2018). Electron microscopy and super-resolution techniques revealed that PML NB typically form a phase-separated spherical shell that tethers to chromatin (Hoischen et al., 2018; Corpet et al., 2020). The PML NB shell results from self-oligomerization that occurs through two steps; 1) the N-terminal RBCC domain of PML primarily facilitates self-oligomerization between PML monomers (Sahin et al., 2014) and 2) further multimerization promoted by PML post-translational modification by SUMO (Hoischen et al., 2018). The PML protein contains at least seven validated SUMOylation sites and a C-terminal SUMO-interacting motif (SIM). This allows SUMO-modified PML proteins to homo-multimerize through their SIM motifs (Song et al., 2004; Cheng and Kao, 2012). Furthermore, many PML NB client proteins associate with PML NBs in a SUMOylation- and/or SIM-dependent manner. For example, two well established PML NB interactors that consistently localize and comprise the “classical” PML NBs are DAXX (via SIMs) and the SP100 (via SUMO) nuclear antigen (Szostecki et al., 1990; Khelifi et al., 2005) (Figure 3). Such SUMO-SIM interactions between partner proteins are fundamental to how classical PML NBs interact with other protein constituents, highlighting the importance of SUMO-SIM interactions in PML biology.

### Novel Promyelocytic Leukemia Containing Nuclear Structures

Although the protein composition of PML NBs is dynamic, the PML NBs themselves are stable structures. However, PML NBs are more dynamic in stressed cells and can shift in morphology and function in the context of cell cycle progression, stressful stimuli and virus infection. There are several non-canonical PML structures that only occur in abnormal cells (Table 1 and Figure 3). For example, in ∼15% of all cancers, cells circumvent their rapid proliferation and eventual loss of telomeres through a pathway known as the alternative lengthening of telomeres (ALT) (Bryan et al., 1995). In ALT-positive cells, PML co-localizes with the telomere marker TRF2 and promotes ALT processes at the telomere (Loe et al., 2020). These novel PML structures, termed ALT-associated PML bodies, are essential for facilitating telomere lengthening by recruiting Bloom syndrome protein (BLM) via SUMO-SIM interactions, and ultimately promoting BLM-TOPO3a-RMI complex formation at telomeres (Zhu et al., 2008; Chung et al., 2012). Therefore, while ALT-associated PML bodies have novel functions, mechanistically their roles are driven by SUMO-SIM interactions as with classical PML NB.

Novel PML structures have also been linked to aging and disease. In senescent cells, PML can appear to form thread-like structures with the nuclear lamina and proteinaceous rings or caps around the nucleoli (Figure 3) (Condemine et al., 2007; Jul-Larsen et al., 2010; Stixova et al., 2012). These structures retain a similar composition to classical PML NBs, harboring DAXX and SP100 (Condemine et al., 2007; Jul-Larsen et al., 2010). Senescence-associated PML-lamina threads occur in Hutchinson-Gilford progeria syndrome (HGPS), a premature aging syndrome resulting from disruption of nuclear lamina integrity (Wang et al., 2020). PML-II is essential for the formation of these PML-lamina threads, which then contribute to the accelerated senescence in HGPS (Jul-Larsen et al., 2010).

While detrimental in HGPS, in other contexts, PML has been shown to help prevent human disease. Neurodegenerative polyglutamine (polyQ) diseases are caused by the expansion of polyQ repeats that are deleterious to neurons (Sato et al., 1999; Yoshizawa et al., 2000). A common pathological feature is the appearance of ubiquitin-positive nuclear inclusions (Qin et al., 2006). To prevent the occurrence of nuclear inclusions and maintain nuclear protein quality, the cell has quality control pathways which include the polyQ-associated PML bodies called PML clastosomes (Figure 3) (Gartner and Muller, 2014). PML is capable of selectively interacting with misfolded nuclear proteins via CRMP-5-associated GTPase (CRAG) through distinct conjugate sites (Guo et al., 2014). Once polyQ misfolded proteins interact with PML NBs, they are conjugated to SUMO, recognized by ring-finger protein 4 (RNF4) and ubiquitinated for degradation through the ubiquitin-proteasome system (Guo et al., 2014). Therefore, PML dynamics in human disease is complex, with novel PML NB structures both promoting and preventing pathologies.

During mitosis, PML proteins form a unique SUMO-negative structure known as mitotic accumulations of PML protein (MAPP) (Figure 3) (Dellaire et al., 2006b). MAPPs also lack DAXX and SP100, which diffuse into the mitotic cytoplasm from prophase to metaphase (Everett et al., 1999; Dellaire et al., 2006b; Chen et al., 2008). MAPPs form once PML NBs are untethered from chromatin during mitosis, and they appear to be bound to early endosomes in dividing cells (Chen et al., 2008; Palibrk et al., 2014). MAPPs remain in the cytoplasm until early G1 phase until the PML protein within them is imported in the nucleus to re-establish PML NBs after the NE reforms (Dellaire et al., 2006b; Chen et al., 2008).

### Lipid-Associated Promyelocytic Leukemia Structures

The recently characterized LAPS represent a new PML subnuclear compartment that associates with nLDs. Most LAPS (∼75%) share similarities to the MAPPs in the sense that they lack SUMO, DAXX and SP100 (Lee et al., 2020). However, LAPS appear to be sphere-like structures, much like classical PML NBs, that form in the nucleus during interphase in response to nLD accumulation. The sequence of events that lead to PML association with nLDs and formation of LAPS has not been fully elucidated. However, in the case of hepatocytes it is mediated by PML-II, which has a unique C-terminal nuclear periphery binding motif that facilitates association with the INM (Figure 1). It is not understood if the interaction of PML-II with the INM is direct, involves adaptor proteins or post-translational modifications (Jul-Larsen et al., 2010). In Huh7 cells, eLDs emerge into the nucleoplasm from the lumen of type-1 NR by membrane disruption at sites of PML-II enrichment and/or depletion of the nuclear lamina, SUN1 and LBR (Figure 1) (Ohsaki et al., 2016; Soltysik et al., 2019). Depletion of PML-II by siRNA knockdown reduced total nLDs by 30–50%, while knockdown of SUN1 and REEP3/4 increased nLDs. The result suggested a model in which PML-II links nLDs to chromatin and the INM that would be otherwise be removed by SUN1 and REEP3/4 (Ohsaki et al., 2016). However, it is unclear how eLDs break through the INM at PML-II patches, and how and when PML-II and other PML isoforms associate with the primordial nLD to form a LAPS.

RNA-mediated silencing of PML-II in U2OS cells did not significantly affect total nLD abundance and size (Soltysik et al., 2021) indicating that nLDs derive from the INM by a mechanism that does not involve PML-II. However, ablation of LAPS by CRISPR/Cas9-mediated PML knockout in U2OS cells significantly reduced nLD abundance by 50% and decreased nLD size (Lee et al., 2020). This discrepancy suggests that a total PML knockout places more stringent conditions on nLD formation and other PML isoforms are involved in LAPS formation. The association of PML with nLDs appears to be dynamic as well, with about 25% of the LAPS resembling classical PML NB containing SP100, SUMO, DAXX, with the remainder being devoid of these canonical partners (Lee et al., 2020). One interpretation is that classical PML NBs initially associate with newly forming nLDs and are gradually remodelled into LAPS as the nLD matures and grows. Most strikingly, this remodeling of PML NBs into LAPS involves loss of SUMO in the structure (Lee et al., 2020). Whether this loss of SUMOylation at LAPS is necessary for the maturation and processing of nLDs is not known, particularly as both yeast and worms form nLDs and lack a PML ortholog (as discussed previously). Since SUMO-SIM interactions play such an important role in PML NB-mediated gene regulation, it would be surprising if such gross reorganization of PML NBs into LAPS would not have some impact on the cell beyond nLD metabolism.

High resolution imaging of immunostained LAPS in U2OS cell revealed that CCTα and PML occupy different regions of the LAPS and have minimal overlap. Clues as to how CCTα and PML form this association with LAPS comes from HGPS fibroblasts in which CCTα is associated with nuclear lamina threads like those observed for PML (Gehrig and Ridgway, 2011). These thread-like PML-II and CCTα structures are likely formed at the type-I NR since CCTα forms foci at normal prelamin-A induced nucleoplasmic reticulum (Goulbourne et al., 2011). In the context of progeria where prelamin-A (progerin) accumulates, this shifts normal foci formation to thread-like structures containing CCTα and PML. Therefore, the prelamin-A type-I NR appears to be a key nucleation center for CCTα and PML foci formation, which could contribute to how PML-coated nLDs are formed. The cell type- and context-specific association of PML-II with the INM supports the idea that additional lipid or protein factors are involved in regulating its membrane association.

The association of PML and nLDs represents a novel function for PML in regulating lipid metabolism through direct structural association with a LD. However, PML and PML NBs have been implicated in lipid metabolism through their more well-established role as gene regulators. PML has been implicated in SREBP signalling (Chen et al., 2018). SREBP-1 and -2, transcription factors that control the expression of genes involved in fatty acid and cholesterol synthesis, are proteolytically processed to their mature nuclear form in response to changes in ER lipid and cholesterol composition (Horton et al., 2003; Brown et al., 2018). In a mouse prostate cancer model, the deletion of *Pml* in *Pten*-null tumours resulted in the hyperactivation of SREBP and ultimately, in a SREBP-dependent aggressive metastatic phenotype (Chen et al., 2018). Mechanistically, this could be related to SREBP-2, which localized to classical PML NBs unlike the diffusely nuclear SREBP-1 (Zoumi et al., 2005). In contrast, PML has an uncharacteristic pro-tumor function in triple negative breast cancers by promoting fatty acid oxidation (Carracedo et al., 2012). Mechanistically, this is accomplished through negative regulation of PGC1A acetylation, likely at PML NBs, which in turn activates PPAR signaling and fatty acid oxidation (Carracedo et al., 2012). Given the ability of SREBP-2 and PGC1A to associate with PML NBs and the presence of Lipin-1─ a SREBP repressor found at PML-containing LAPS─ it seems plausible that LAPS might affect SREBP activity. Despite this intriguing hypothesis, to date SREBP-2 and PGC1A localization to LAPS has not been evaluated. Taken together, the results suggest that LAPS put PML as a physical interactor and contributor to nLD formation, and therefore lipid metabolism under conditions of fatty acid stress in mammals. However, there are also roles for the canonical PML structures in regulating lipid metabolism in other contexts via SREBPs and fatty acid oxidation that most likely contribute to lipid-stress responses.

## Conclusion

nLDs are emerging as potentially important organelles in their capacity to; 1) store and supply lipid precursors for membrane biogenesis and signalling molecules in the nucleus, and 2) regulate the cellular response to fatty acid stress. Although two different pathways for nLD biogenesis have been identified, both involve maturation by the *de novo* synthesis of TAG at the INM or on nLDs. Regardless of the mechanism of biogenesis, newly formed nLDs are an important regulatory site for PC synthesis via recruitment and activation of CCTα to enhance the TAG storage capacity or relieve ER stress. However, many questions remain, such as why do nLDs only appear in certain cell types and species, how are lipids in nLDs degraded and/or redistributed in the nucleus, how are cytosolic lipid metabolic enzymes imported into the nucleus to make nLDs and how are these enzymes recruited to the INM and nLDs?

Studies of the nLD maturation process has also revealed an important role for PML, and particularly PML-II, in the formation of a discrete subset of nLDs called LAPS. LAPS represent a novel PML-containing subnuclear structure that adds to our understanding of how the PML protein responds to various cellular stresses and disease states by altering the structure, composition, and localization of PML NBs. In the case of LAPS, there is much work to be in done to fully elucidate what role(s) PML might play in LAPS biology beyond LAPS formation and the recruitment of CCTα and Lipin-1. The presence of these lipid enzymes and the concurrent loss of SUMO, SP100 and DAXX from LAPS indicates they are unique PML-containing structures specialized in regulating lipid metabolism. It will be important to identify the proteome of nLDs and the role that PML plays in the association of client proteins with LAPS. In the absence of SUMO-SIM interactions, which drive the majority of protein associations with PML NBs, one might expect a more limited and specific repertoire of LAPS-associated proteins that rely on conventional protein-protein or protein-lipid interactions to be recruited and retained at LAPS. In this situation it is possible that specific PML isoforms, through their unique C-terminal regions, could recruit LAPS-associated proteins in a more direct way. Alternatively, or in addition to, PML-containing portion of LAPS could retain the phase separation properties of PML NBs to recruit and retain proteins in liquid condensates. In addition, it is still unclear if LAPS are associated with chromatin regions or specific gene promoters in the same way as PML NBs, although we do know that nLDs can modify nuclear architecture and chromatin dynamics. Therefore, PML association with nLDs through LAPS formation might provide a mechanism for the cell to effect transcriptional changes as part of the lipid stress response in much the same way that PML NBs regulate gene expression through chromatin contacts as well as recruitment and modification of transcription factors and chromatin modifying enzymes. The lack of key PML NB components at LAPS would, however, indicate that the mechanism for gene regulation at LAPS involves a different complement of PML-associated proteins that do not interact with LAPS via SUMO-SIM interactions. Collectively, these findings usher in new paradigms for not only PML-based nuclear structures but also for nLD biology.

## Abbreviations

AGPAT, 1-acylglycerol 3-phosphate acyltransferases; ALT, alternative lengthening of telomeres; BLM, Bloom syndrome protein cLD, cytoplasmic lipid droplets; CEPT, choline/ethanolamine phosphotransferase; CPT, choline phosphotransferase; CRAG, CRMP-5-associated GTPase; CCTα, CTP:phosphocholine cytidylyltransferase α; DAG, diacylglycerol; DGAT, DAG acyltransferase; DAXX, Death domain-associated protein 6; ER, endoplasmic reticulum; eLD, ER luminal lipid droplets; GPAT; glycerol 3-phosphate acyltransferases; HGPS, Hutchinson-Gilford progeria syndrome INM, inner nuclear membrane; LAPS, lipid-associated PML structures; LD, lipid droplets; MAPP, mitotic accumulations of PML protein; MTP, microsomal triglyceride transfer protein; NAFLD, non-alcoholic fatty liver disease; NR, nucleoplasmic reticulum; NE, nuclear envelope; nLD, nuclear lipid droplets; PA, phosphatidic acid; PGC1A, PPARγ co-activator 1A; PML, promyelocytic leukemia; PPAR, peroxisome proliferator-activated receptors; RBCC, RING-B-box-coiled-coiled; SUMO, small-ubiquitin like modifier; SIM, SUM-interaction motif; SREBP, sterol regulatory element-binding protein; ring finger protein 4, RNF5; TAG, triacylglyceride; VLDL, very-low density lipoproteins.

## References

[B1] Aitchison A. J. Arsenault D. J. Ridgway N. D. (2015). Nuclear-localized CTP:phosphocholine Cytidylyltransferase α Regulates Phosphatidylcholine Synthesis Required for Lipid Droplet Biogenesis. MBoC 26, 2927–2938. 10.1091/mbc.e15-03-0159 26108622 PMC4571330

[B2] Attwood K. M. Salsman J. Chung D. Mathavarajah S. Van Iderstine C. Dellaire G. (2020). PML Isoform Expression and DNA Break Location Relative to PML Nuclear Bodies Impacts the Efficiency of Homologous Recombination. Biochem. Cel Biol. 98, 314–326. 10.1139/bcb-2019-0115 31671275

[B3] Banani S. F. Rice A. M. Peeples W. B. Lin Y. Jain S. Parker R. (2016). Compositional Control of Phase-Separated Cellular Bodies. Cell 166, 651–663. 10.1016/j.cell.2016.06.010 27374333 PMC4967043

[B4] Barger S. R. Penfield L. Bahmanyar S. (2022). Coupling Lipid Synthesis with Nuclear Envelope Remodeling. Trends Biochem. Sci. 47, 52–65. 10.1016/j.tibs.2021.08.009 34556392 PMC9943564

[B5] Barreyro F. J. Kobayashi S. Bronk S. F. Werneburg N. W. Malhi H. Gores G. J. (2007). Transcriptional Regulation of Bim by FoxO3A Mediates Hepatocyte Lipoapoptosis. J. Biol. Chem. 282, 27141–27154. 10.1074/jbc.m704391200 17626006

[B6] Barroso-Gomila O. Trulsson F. Muratore V. Canosa I. Merino-Cacho L. Cortazar A. R. (2021). Identification of Proximal SUMO-dependent Interactors Using SUMO-ID. Nat. Commun. 12, 6671. 10.1038/s41467-021-26807-6 34795231 PMC8602451

[B7] Bernardi R. Papa A. Pandolfi P. P. (2008). Regulation of Apoptosis by PML and the PML-NBs. Oncogene 27, 6299–6312. 10.1038/onc.2008.305 18931695

[B8] Bischof O. Kirsh O. Pearson M. Itahana K. Pelicci P. G. Dejean A. (2002). Deconstructing PML-Induced Premature Senescence. EMBO J. 21, 3358–3369. 10.1093/emboj/cdf341 12093737 PMC126090

[B9] Bosch M. Sweet M. J. Parton R. G. Pol A. (2021). Lipid Droplets and the Host-Pathogen Dynamic: FATal Attraction? J. Cel Biol. 220, e202104005. 10.1083/jcb.202104005 PMC8240858 34165498

[B10] Brown J. R. Conn K. L. Wasson P. Charman M. Tong L. Grant K. (2016). SUMO Ligase Protein Inhibitor of Activated STAT1 (PIAS1) Is a Constituent Promyelocytic Leukemia Nuclear Body Protein that Contributes to the Intrinsic Antiviral Immune Response to Herpes Simplex Virus 1. J. Virol. 90, 5939–5952. 10.1128/jvi.00426-16 27099310 PMC4907222

[B11] Brown M. S. Radhakrishnan A. Goldstein J. L. (2018). Retrospective on Cholesterol Homeostasis: The Central Role of Scap. Annu. Rev. Biochem. 87, 783–807. 10.1146/annurev-biochem-062917-011852 28841344 PMC5828883

[B12] Bryan T. M. Englezou A. Gupta J. Bacchetti S. Reddel R. R. (1995). Telomere Elongation in Immortal Human Cells without Detectable Telomerase Activity. EMBO J. 14, 4240–4248. 10.1002/j.1460-2075.1995.tb00098.x 7556065 PMC394507

[B13] Carman G. M. Han G.-S. (2011). Regulation of Phospholipid Synthesis in the Yeast *Saccharomyces cerevisiae* . Annu. Rev. Biochem. 80, 859–883. 10.1146/annurev-biochem-060409-092229 21275641 PMC3565220

[B14] Carracedo A. Weiss D. Leliaert A. K. Bhasin M. de Boer V. C. J. Laurent G. (2012). A Metabolic Prosurvival Role for PML in Breast Cancer. J. Clin. Invest. 122, 3088–3100. 10.1172/jci62129 22886304 PMC3433768

[B15] Cazanave S. C. Elmi N. A. Akazawa Y. Bronk S. F. Mott J. L. Gores G. J. (2010). CHOP and AP-1 Cooperatively Mediate PUMA Expression during Lipoapoptosis. Am. J. Physiol. Gastrointestinal Liver Physiol. 299, G236–G243. 10.1152/ajpgi.00091.2010 PMC2904106 20430872

[B16] Cermelli S. Guo Y. Gross S. P. Welte M. A. (2006). The Lipid-Droplet Proteome Reveals that Droplets Are a Protein-Storage Depot. Curr. Biol. 16, 1783–1795. 10.1016/j.cub.2006.07.062 16979555

[B17] Chen Y.-C. M. Kappel C. Beaudouin J. Eils R. Spector D. L. (2008). Live Cell Dynamics of Promyelocytic Leukemia Nuclear Bodies upon Entry into and Exit from Mitosis. MBoC 19, 3147–3162. 10.1091/mbc.e08-01-0035 18480407 PMC2441680

[B18] Chen Z. Gropler M. C. Mitra M. S. Finck B. N. (2012). Complex Interplay between the Lipin 1 and the Hepatocyte Nuclear Factor 4 α (HNF4α) Pathways to Regulate Liver Lipid Metabolism. PLoS One 7, e51320. 10.1371/journal.pone.0051320 23236470 PMC3517414

[B19] Chen M. Zhang J. Sampieri K. Clohessy J. G. Mendez L. Gonzalez-Billalabeitia E. (2018). An Aberrant SREBP-dependent Lipogenic Program Promotes Metastatic Prostate Cancer. Nat. Genet. 50, 206–218. 10.1038/s41588-017-0027-2 29335545 PMC6714980

[B20] Cheng X. Kao H. Y. (2012). Post-translational Modifications of PML: Consequences and Implications. Front. Oncol. 2, 210. 10.3389/fonc.2012.00210 23316480 PMC3539660

[B21] Chung I. Osterwald S. Deeg K. I. Rippe K. (2012). PML Body Meets Telomere. Nucleus 3, 263–275. 10.4161/nucl.20326 22572954 PMC3414403

[B22] Chung J. Wu X. Lambert T. J. Lai Z. W. Walther T. C. Farese R. V. Jr. (2019). LDAF1 and Seipin Form a Lipid Droplet Assembly Complex. Develop. Cel 51, 551–563. 10.1016/j.devcel.2019.10.006 PMC7235935 31708432

[B23] Condemine W. Takahashi Y. Le Bras M. de Thé H. (2007). A Nucleolar Targeting Signal in PML-I Addresses PML to Nucleolar Caps in Stressed or Senescent Cells. J. Cel Sci. 120, 3219–3227. 10.1242/jcs.007492 17878236

[B24] Cornell R. B. Ridgway N. D. (2015). CTP:phosphocholine Cytidylyltransferase: Function, Regulation, and Structure of an Amphitropic Enzyme Required for Membrane Biogenesis. Prog. Lipid Res. 59, 147–171. 10.1016/j.plipres.2015.07.001 26165797

[B25] Corpet A. Olbrich T. Gwerder M. Fink D. Stucki M. (2014). Dynamics of Histone H3.3 Deposition in Proliferating and Senescent Cells Reveals a DAXX-dependent Targeting to PML-NBs Important for Pericentromeric Heterochromatin Organization. Cell Cycle 13, 249–267. 10.4161/cc.26988 24200965 PMC3906242

[B26] Corpet A. Kleijwegt C. Roubille S. Juillard F. Jacquet K. Texier P. (2020). PML Nuclear Bodies and Chromatin Dynamics: Catch Me if You Can! Nucleic Acids Res. 48, 11890–11912. 10.1093/nar/gkaa828 33068409 PMC7708061

[B27] Csaki L. S. Dwyer J. R. Fong L. G. Tontonoz P. Young S. G. Reue K. (2013). Lipins, Lipinopathies, and the Modulation of Cellular Lipid Storage and Signaling. Prog. Lipid Res. 52, 305–316. 10.1016/j.plipres.2013.04.001 23603613 PMC3830937

[B28] Dechat T. Pfleghaar K. Sengupta K. Shimi T. Shumaker D. K. Solimando L. (2008). Nuclear Lamins: Major Factors in the Structural Organization and Function of the Nucleus and Chromatin. Genes Dev. 22, 832–853. 10.1101/gad.1652708 18381888 PMC2732390

[B29] Dellaire G. Bazett-Jones D. P. (2004). PML Nuclear Bodies: Dynamic Sensors of DNA Damage and Cellular Stress. Bioessays 26, 963–977. 10.1002/bies.20089 15351967

[B30] Dellaire G. Ching R. W. Ahmed K. Jalali F. Tse K. C. K. Bristow R. G. (2006a). Promyelocytic Leukemia Nuclear Bodies Behave as DNA Damage Sensors Whose Response to DNA Double-Strand Breaks Is Regulated by NBS1 and the Kinases ATM, Chk2, and ATR. J. Cel Biol. 175, 55–66. 10.1083/jcb.200604009 PMC2064496 17030982

[B31] Dellaire G. Eskiw C. H. Dehghani H. Ching R. W. Bazett-Jones D. P. (2006b). Mitotic Accumulations of PML Protein Contribute to the Re-establishment of PML Nuclear Bodies in G1. J. Cel Sci. 119, 1034–1042. 10.1242/jcs.02817 16492707

[B32] Diehl A. M. Day C. (2017). Cause, Pathogenesis, and Treatment of Nonalcoholic Steatohepatitis. N. Engl. J. Med. 377, 2063–2072. 10.1056/nejmra1503519 29166236

[B33] Drané P. Ouararhni K. Depaux A. Shuaib M. Hamiche A. (2010). The Death-Associated Protein DAXX Is a Novel Histone Chaperone Involved in the Replication-independent Deposition of H3.3. Genes Dev. 24, 1253–1265. 10.1101/gad.566910 20504901 PMC2885661

[B34] Drozdz M. M. Jiang H. Pytowski L. Grovenor C. Vaux D. J. (2017). Formation of a Nucleoplasmic Reticulum Requires De Novo Assembly of Nascent Phospholipids and Shows Preferential Incorporation of Nascent Lamins. Sci. Rep. 7, 7454. 10.1038/s41598-017-07614-w 28785031 PMC5547041

[B35] Du X. Barisch C. Paschke P. Herrfurth C. Bertinetti O. Pawolleck N. (2013). Dictyostelium Lipid Droplets Host Novel Proteins. Eukaryot. Cel 12, 1517–1529. 10.1128/ec.00182-13 PMC3837934 24036346

[B36] Everett R. D. Lomonte P. Sternsdorf T. van Driel R. Orr A. (1999). Cell Cycle Regulation of PML Modification and ND10 Composition. J. Cel Sci. 112 (Pt 24), 4581–4588. 10.1242/jcs.112.24.4581 10574707

[B37] Finck B. N. Gropler M. C. Chen Z. Leone T. C. Croce M. A. Harris T. E. (2006). Lipin 1 Is an Inducible Amplifier of the Hepatic PGC-1α/PPARα Regulatory Pathway. Cel Metab. 4, 199–210. 10.1016/j.cmet.2006.08.005 16950137

[B38] Gärtner A. Muller S. (2014). PML, SUMO, and RNF4: Guardians of Nuclear Protein Quality. Mol. Cel 55, 1–3. 10.1016/j.molcel.2014.06.022 24996060

[B39] Gehrig K. Ridgway N. D. (2011). CTP:phosphocholine Cytidylyltransferase α (CCTα) and Lamins Alter Nuclear Membrane Structure without Affecting Phosphatidylcholine Synthesis. Biochim. Biophys. Acta Mol. Cel Biol. Lipids 1811, 377–385. 10.1016/j.bbalip.2011.04.001 21504799

[B40] Gehrig K. Cornell R. B. Ridgway N. D. (2008). Expansion of the Nucleoplasmic Reticulum Requires the Coordinated Activity of Lamins and CTP:Phosphocholine Cytidylyltransferase α. MBoC 19, 237–247. 10.1091/mbc.e07-02-0179 17959832 PMC2174170

[B41] Goulbourne C. N. Malhas A. N. Vaux D. J. (2011). The Induction of a Nucleoplasmic Reticulum by Prelamin A Accumulation Requires CTP:phosphocholine Cytidylyltransferase-α. J. Cel Sci. 124, 4253–4266. 10.1242/jcs.091009 PMC3258109 22223883

[B42] Grillet M. Dominguez Gonzalez B. Sicart A. Pöttler M. Cascalho A. Billion K. (2016). Torsins Are Essential Regulators of Cellular Lipid Metabolism. Develop. Cel 38, 235–247. 10.1016/j.devcel.2016.06.017 27453503

[B43] Guo L. Giasson B. I. Glavis-Bloom A. Brewer M. D. Shorter J. Gitler A. D. (2014). A Cellular System that Degrades Misfolded Proteins and Protects against Neurodegeneration. Mol. Cel 55, 15–30. 10.1016/j.molcel.2014.04.030 PMC4445634 24882209

[B44] Gushchina L. V. Kwiatkowski T. A. Bhattacharya S. Weisleder N. L. (2018). Conserved Structural and Functional Aspects of the Tripartite Motif Gene Family point towards Therapeutic Applications in Multiple Diseases. Pharmacol. Ther. 185, 12–25. 10.1016/j.pharmthera.2017.10.020 29097306 PMC5721676

[B45] Hattersley N. Shen L. Jaffray E. G. Hay R. T. (2011). The SUMO Protease SENP6 Is a Direct Regulator of PML Nuclear Bodies. MBoC 22, 78–90. 10.1091/mbc.e10-06-0504 21148299 PMC3016979

[B46] Henne W. M. Reese M. L. Goodman J. M. (2018). The Assembly of Lipid Droplets and Their Roles in Challenged Cells. EMBO J. 37, e98947. 10.15252/embj.201898947 29789390 PMC6003646

[B47] Henneberry A. L. Wright M. M. McMaster C. R. (2002). The Major Sites of Cellular Phospholipid Synthesis and Molecular Determinants of Fatty Acid and Lipid Head Group Specificity. MBoC 13, 3148–3161. 10.1091/mbc.01-11-0540 12221122 PMC124149

[B48] Hoischen C. Monajembashi S. Weisshart K. Hemmerich P. (2018). Multimodal Light Microscopy Approaches to Reveal Structural and Functional Properties of Promyelocytic Leukemia Nuclear Bodies. Front. Oncol. 8, 125. 10.3389/fonc.2018.00125 29888200 PMC5980967

[B49] Horton J. D. Shah N. A. Warrington J. A. Anderson N. N. Park S. W. Brown M. S. (2003). Combined Analysis of Oligonucleotide Microarray Data from Transgenic and Knockout Mice Identifies Direct SREBP Target Genes. Proc. Natl. Acad. Sci. 100, 12027–12032. 10.1073/pnas.1534923100 14512514 PMC218707

[B122] Imrichova T. Hubackova A. Kucerova A. Kosla J. Bartek Z. Hodny J. (2019). Dynamic PML Protein Nucleolar Associations With Persistent DNA Damage Lesions in Response to Nucleolar Stress and Senescence-Inducing Stimuli. Aging 11, 7206–7235. 31493766 10.18632/aging.102248 PMC6756913

[B50] Jacquemyn J. Foroozandeh J. Vints K. Swerts J. Verstreken P. Gounko N. V. (2021). Torsin and NEP1R1-CTDNEP1 Phosphatase Affect Interphase Nuclear Pore Complex Insertion by Lipid-dependent and Lipid-independent Mechanisms. EMBO J. 40, e106914. 10.15252/embj.2020106914 34313336 PMC8408595

[B123] Janer A. Martin E. Muriel M. P. Latouche M. Fujigasaki H. Ruberg M. (2006). PML Clastosomes Prevent Nuclear Accumulation of Mutant Ataxin-7 and Other Polyglutamine Proteins. J. Cell Biol. 174, 65–76. 16818720 10.1083/jcb.200511045 PMC2064165

[B51] Janowski B. A. Willy P. J. Devi T. R. Falck J. R. Mangelsdorf D. J. (1996). An Oxysterol Signalling Pathway Mediated by the Nuclear Receptor LXRα. Nature 383, 728–731. 10.1038/383728a0 8878485

[B52] Jensen K. Shiels C. Freemont P. S. (2001). PML Protein Isoforms and the RBCC/TRIM Motif. Oncogene 20, 7223–7233. 10.1038/sj.onc.1204765 11704850

[B53] Jul-Larsen A. Grudic A. Bjerkvig R. Bøe S. O. (2010). Subcellular Distribution of Nuclear Import-Defective Isoforms of the Promyelocytic Leukemia Protein. BMC Mol. Biol. 11, 89. 10.1186/1471-2199-11-89 21092142 PMC2998510

[B54] Khelifi A. F. D'Alcontres M. S. Salomoni P. (2005). Daxx Is Required for Stress-Induced Cell Death and JNK Activation. Cell Death Differ. 12, 724–733. 10.1038/sj.cdd.4401559 15861194

[B55] Kornke J. M. Maniak M. (2017). Fat-containing Cells Are Eliminated during Dictyostelium Development. Biol. Open 6, 1294–1304. 10.1242/bio.025478 28751309 PMC5612234

[B56] Krahmer N. Guo Y. Wilfling F. Hilger M. Lingrell S. Heger K. (2011). Phosphatidylcholine Synthesis for Lipid Droplet Expansion Is Mediated by Localized Activation of CTP:phosphocholine Cytidylyltransferase. Cel Metab. 14, 504–515. 10.1016/j.cmet.2011.07.013 PMC3735358 21982710

[B57] Kumanski S. Viart B. Kossida S. Moriel-Carretero M. (2021). Lipid Droplets Are a Physiological Nucleoporin Reservoir. Cells 10, 472–481. 10.3390/cells10020472 33671805 PMC7926788

[B58] Lagace T. A. Ridgway N. D. (2005). The Rate-Limiting Enzyme in Phosphatidylcholine Synthesis Regulates Proliferation of the Nucleoplasmic Reticulum. MBoC 16, 1120–1130. 10.1091/mbc.e04-10-0874 15635091 PMC551478

[B59] Lagrutta L. C. Layerenza J. P. Bronsoms S. Trejo S. A. Ves-Losada A. (2021). Nuclear-lipid-droplet Proteome: Carboxylesterase as a Nuclear Lipase Involved in Lipid-Droplet Homeostasis. Heliyon 7, e06539. 10.1016/j.heliyon.2021.e06539 33817385 PMC8010399

[B60] Layerenza J. P. González P. García de Bravo M. M. Polo M. P. Sisti M. S. Ves-Losada A. (2013). Nuclear Lipid Droplets: a Novel Nuclear Domain. Biochim. Biophys. Acta Mol. Cel Biol. Lipids 1831, 327–340. 10.1016/j.bbalip.2012.10.005 23098923

[B61] Lee J. Salsman J. Foster J. Dellaire G. Ridgway N. D. (2020). Lipid-associated PML Structures Assemble Nuclear Lipid Droplets Containing CCTα and Lipin1. Life Sci. Alliance 3, e202000751. 10.26508/lsa.202000751 32461215 PMC7266991

[B62] Lehner R. Lian J. Quiroga A. D. (2012). Lumenal Lipid Metabolism. Arterioscler Thromb. Vasc. Biol. 32, 1087–1093. 10.1161/atvbaha.111.241497 22517367

[B63] Li Z. Thiel K. Thul P. J. Beller M. Kühnlein R. P. Welte M. A. (2012). Lipid Droplets Control the Maternal Histone Supply of Drosophila Embryos. Curr. Biol. 22, 2104–2113. 10.1016/j.cub.2012.09.018 23084995 PMC3513403

[B64] Liu Y. Xu S. Zhang C. Zhu X. Hammad M. A. Zhang X. (2018). Hydroxysteroid Dehydrogenase Family Proteins on Lipid Droplets through Bacteria, *C. elegans*, and Mammals. Biochim. Biophys. Acta Mol. Cel Biol. Lipids 1863, 881–894. 10.1016/j.bbalip.2018.04.018 29702244

[B65] Loe T. K. Li J. S. Z. Zhang Y. Azeroglu B. Boddy M. N. Denchi E. L. (2020). Telomere Length Heterogeneity in ALT Cells Is Maintained by PML-dependent Localization of the BTR Complex to Telomeres. Genes Dev. 34, 650–662. 10.1101/gad.333963.119 32217664 PMC7197349

[B66] Mantzaris M. D. Tsianos E. V. Galaris D. (2011). Interruption of Triacylglycerol Synthesis in the Endoplasmic Reticulum Is the Initiating Event for Saturated Fatty Acid-Induced Lipotoxicity in Liver Cells. FEBS J. 278, 519–530. 10.1111/j.1742-4658.2010.07972.x 21182590

[B67] McFie P. J. Banman S. L. Stone S. J. (2018). Diacylglycerol Acyltransferase-2 Contains a C-Terminal Sequence that Interacts with Lipid Droplets. Biochim. Biophys. Acta Mol. Cel Biol. Lipids 1863, 1068–1081. 10.1016/j.bbalip.2018.06.008 29902571

[B68] Mejhert N. Kuruvilla L. Gabriel K. R. Elliott S. D. Guie M.-A. Wang H. (2020). Partitioning of MLX-Family Transcription Factors to Lipid Droplets Regulates Metabolic Gene Expression. Mol. Cel 77, 1251–1264. 10.1016/j.molcel.2020.01.014 PMC7397554 32023484

[B69] Moldavski O. Amen T. Levin-Zaidman S. Eisenstein M. Rogachev I. Brandis A. (2015). Lipid Droplets Are Essential for Efficient Clearance of Cytosolic Inclusion Bodies. Develop. Cel 33, 603–610. 10.1016/j.devcel.2015.04.015 26004510

[B70] Mosquera J. V. Bacher M. C. Priess J. R. (2021). Nuclear Lipid Droplets and Nuclear Damage in *Caenorhabditis elegans* . Plos Genet. 17, e1009602. 10.1371/journal.pgen.1009602 34133414 PMC8208577

[B71] Musille P. M. Pathak M. C. Lauer J. L. Hudson W. H. Griffin P. R. Ortlund E. A. (2012). Antidiabetic Phospholipid-Nuclear Receptor Complex Reveals the Mechanism for Phospholipid-Driven Gene Regulation. Nat. Struct. Mol. Biol. 19, 532–537. 10.1038/nsmb.2279 22504882 PMC3960984

[B72] Nisole S. Maroui M. A. Mascle X. H. Aubry M. Chelbi-Alix M. K. (2013). Differential Roles of PML Isoforms. Front. Oncol. 3, 125. 10.3389/fonc.2013.00125 23734343 PMC3660695

[B73] Ohsaki Y. Cheng J. Suzuki M. Fujita A. Fujimoto T. (2008). Lipid Droplets Are Arrested in the ER Membrane by Tight Binding of Lipidated Apolipoprotein B-100. J. Cel Sci. 121, 2415–2422. 10.1242/jcs.025452 18577578

[B74] Ohsaki Y. Kawai T. Yoshikawa Y. Cheng J. Jokitalo E. Fujimoto T. (2016). PML Isoform II Plays a Critical Role in Nuclear Lipid Droplet Formation. J. Cel Biol. 212, 29–38. 10.1083/jcb.201507122 PMC4700481 26728854

[B75] Olarte M.-J. Swanson J. M. J. Walther T. C. Farese R. V. Jr. (2022). The CYTOLD and ERTOLD Pathways for Lipid Droplet-Protein Targeting. Trends Biochem. Sci. 47, 39–51. 10.1016/j.tibs.2021.08.007 34583871 PMC8688270

[B76] Olzmann J. A. Carvalho P. (2019). Dynamics and Functions of Lipid Droplets. Nat. Rev. Mol. Cel Biol. 20, 137–155. 10.1038/s41580-018-0085-z PMC6746329 30523332

[B77] Orban T. Palczewska G. Palczewski K. (2011). Retinyl Ester Storage Particles (Retinosomes) from the Retinal Pigmented Epithelium Resemble Lipid Droplets in Other Tissues. J. Biol. Chem. 286, 17248–17258. 10.1074/jbc.m110.195198 21454509 PMC3089567

[B78] Pais R. Barritt A. S. Calmus Y. Scatton O. Runge T. Lebray P. (2016). NAFLD and Liver Transplantation: Current burden and Expected Challenges. J. Hepatol. 65, 1245–1257. 10.1016/j.jhep.2016.07.033 27486010 PMC5326676

[B79] Palibrk V. Lång E. Lång A. Schink K. O. Rowe A. D. Bøe S. O. (2014). Promyelocytic Leukemia Bodies Tether to Early Endosomes during Mitosis. Cell Cycle 13, 1749–1755. 10.4161/cc.28653 24675887 PMC4111721

[B80] Pearson M. Pelicci P. G. (2001). PML Interaction with P53 and its Role in Apoptosis and Replicative Senescence. Oncogene 20, 7250–7256. 10.1038/sj.onc.1204856 11704853

[B81] Perez M. F. Lehner B. (2019). Vitellogenins - Yolk Gene Function and Regulation in *Caenorhabditis elegans* . Front. Physiol. 10, 1067. 10.3389/fphys.2019.01067 31551797 PMC6736625

[B82] Peterson T. R. Sengupta S. S. Harris T. E. Carmack A. E. Kang S. A. Balderas E. (2011). mTOR Complex 1 Regulates Lipin 1 Localization to Control the SREBP Pathway. Cell 146, 408–420. 10.1016/j.cell.2011.06.034 21816276 PMC3336367

[B83] Qin Q. Inatome R. Hotta A. Kojima M. Yamamura H. Hirai H. (2006). A Novel GTPase, CRAG, Mediates Promyelocytic Leukemia Protein-Associated Nuclear Body Formation and Degradation of Expanded Polyglutamine Protein. J. Cel Biol. 172, 497–504. 10.1083/jcb.200505079 PMC2063670 16461359

[B84] Rava P. Hussain M. M. (2007). Acquisition of Triacylglycerol Transfer Activity by Microsomal Triglyceride Transfer Protein during Evolution. Biochemistry 46, 12263–12274. 10.1021/bi700762z 17924655 PMC2536605

[B85] Romanauska A. Köhler A. (2018). The Inner Nuclear Membrane Is a Metabolically Active Territory that Generates Nuclear Lipid Droplets. Cell 174, 700–715. 10.1016/j.cell.2018.05.047 29937227 PMC6371920

[B86] Romanauska A. Köhler A. (2021). Reprogrammed Lipid Metabolism Protects Inner Nuclear Membrane against Unsaturated Fat. Develop. Cel 56, 2562–2578. 10.1016/j.devcel.2021.07.018 PMC8480995 34407429

[B87] Sahin U. Ferhi O. Jeanne M. Benhenda S. Berthier C. Jollivet F. (2014). Oxidative Stress-Induced Assembly of PML Nuclear Bodies Controls Sumoylation of Partner Proteins. J. Cel Biol. 204, 931–945. 10.1083/jcb.201305148 PMC3998805 24637324

[B88] Salo V. T. Li S. Vihinen H. Hölttä-Vuori M. Szkalisity A. Horvath P. (2019). Seipin Facilitates Triglyceride Flow to Lipid Droplet and Counteracts Droplet Ripening via Endoplasmic Reticulum Contact. Develop. Cel 50, 478–493. 10.1016/j.devcel.2019.05.016 31178403

[B89] Sato A. Shimohata T. Koide R. Takano H. Sato T. Oyake M. (1999). Adenovirus-mediated Expression of Mutant DRPLA Proteins with Expanded Polyglutamine Stretches in Neuronally Differentiated PC12 Cells. Preferential Intranuclear Aggregate Formation and Apoptosis. Hum. Mol. Genet. 8, 997–1006. 10.1093/hmg/8.6.997 10332031

[B90] Shen W.-J. Azhar S. Kraemer F. B. (2016). Lipid Droplets and Steroidogenic Cells. Exp. Cel Res. 340, 209–214. 10.1016/j.yexcr.2015.11.024 PMC4744538 26639173

[B91] Shibano T. Mamada H. Hakuno F. Takahashi S.-I. Taira M. (2015). The Inner Nuclear Membrane Protein Nemp1 Is a New Type of RanGTP-Binding Protein in Eukaryotes. PLoS One 10, e0127271. 10.1371/journal.pone.0127271 25946333 PMC4422613

[B92] Shin J.-Y. Hernandez-Ono A. Fedotova T. Östlund C. Lee M. J. Gibeley S. B. (2019). Nuclear Envelope-Localized torsinA-LAP1 Complex Regulates Hepatic VLDL Secretion and Steatosis. J. Clin. Invest. 129, 4885–4900. 10.1172/jci129769 31408437 PMC6819140

[B93] Sim M. F. Dennis R. J. Aubry E. M. Ramanathan N. Sembongi H. Saudek V. (2012). The Human Lipodystrophy Protein Seipin Is an ER Membrane Adaptor for the Adipogenic PA Phosphatase Lipin 1. Mol. Metab. 2, 38–46. 10.1016/j.molmet.2012.11.002 24024128 PMC3757660

[B94] Sim M. F. M. Persiani E. Talukder M. M. U. McIlroy G. D. Roumane A. Edwardson J. M. (2020). Oligomers of the Lipodystrophy Protein Seipin May Co-ordinate GPAT3 and AGPAT2 Enzymes to Facilitate Adipocyte Differentiation. Sci. Rep. 10, 3259. 10.1038/s41598-020-59982-5 32094408 PMC7039881

[B95] Singh R. Kaushik S. Wang Y. Xiang Y. Novak I. Komatsu M. (2009). Autophagy Regulates Lipid Metabolism. Nature 458, 1131–1135. 10.1038/nature07976 19339967 PMC2676208

[B96] Smoyer C. J. Katta S. S. Gardner J. M. Stoltz L. McCroskey S. Bradford W. D. (2016). Analysis of Membrane Proteins Localizing to the Inner Nuclear Envelope in Living Cells. J. Cel Biol 215, 575–590. 10.1083/jcb.201607043 PMC5119940 27831485

[B97] Sołtysik K. Ohsaki Y. Fujimoto T. (2019). Duo in a Mystical Realm-Nuclear Lipid Droplets and the Inner Nuclear Membrane. Contact 2, 251525641989696. 10.1177/2515256419896965

[B98] Soltysik K. Ohsaki Y. Tatematsu T. Cheng J. Fujimoto T. (2019). Nuclear Lipid Droplets Derive from a Lipoprotein Precursor and Regulate Phosphatidylcholine Synthesis. Nat. Commun. 10, 473. 10.1038/s41467-019-08411-x 30692541 PMC6349838

[B99] Soltysik K. Ohsaki Y. Tatematsu T. Cheng J. Maeda A. Morita S. Y. (2021). Nuclear Lipid Droplets Form in the Inner Nuclear Membrane in a Seipin-independent Manner. J. Cel Biol. 220, e202005026. 10.1083/jcb.202005026 PMC7737703 33315072

[B100] Song J. Durrin L. K. Wilkinson T. A. Krontiris T. G. Chen Y. (2004). Identification of a SUMO-Binding Motif that Recognizes SUMO-Modified Proteins. Proc. Natl. Acad. Sci. 101, 14373–14378. 10.1073/pnas.0403498101 15388847 PMC521952

[B101] Soni K. G. Mardones G. A. Sougrat R. Smirnova E. Jackson C. L. Bonifacino J. S. (2009). Coatomer-dependent Protein Delivery to Lipid Droplets. J. Cel Sci. 122, 1834–1841. 10.1242/jcs.045849 PMC2684835 19461073

[B102] Stixová L. Matula P. Kozubek S. Gombitová A. Cmarko D. Raška I. (2012). Trajectories and Nuclear Arrangement of PML Bodies Are Influenced by A-type Lamin Deficiency. Biol. Cel 104, 418–432. 10.1111/boc.201100053 22443097

[B103] Szostecki C. Guldner H. H. Netter H. J. Will H. (1990). Isolation and Characterization of cDNA Encoding a Human Nuclear Antigen Predominantly Recognized by Autoantibodies from Patients with Primary Biliary Cirrhosis. J. Immunol. 145, 4338–4347. 2258622

[B104] Tanaka N. Aoyama T. Kimura S. Gonzalez F. J. (2017). Targeting Nuclear Receptors for the Treatment of Fatty Liver Disease. Pharmacol. Ther. 179, 142–157. 10.1016/j.pharmthera.2017.05.011 28546081 PMC6659998

[B105] Thiam A. R. Farese Jr R. V. Jr. Walther T. C. (2013). The Biophysics and Cell Biology of Lipid Droplets. Nat. Rev. Mol. Cel Biol. 14, 775–786. 10.1038/nrm3699 PMC4526153 24220094

[B106] Uzbekov R. Roingeard P. (2013). Nuclear Lipid Droplets Identified by Electron Microscopy of Serial Sections. BMC Res. Notes 6, 386. 10.1186/1756-0500-6-386 24070407 PMC3849021

[B107] Van Damme E. Laukens K. Dang T. H. Van Ostade X. (2010). A Manually Curated Network of the PML Nuclear Body Interactome Reveals an Important Role for PML-NBs in SUMOylation Dynamics. Int. J. Biol. Sci. 6, 51–67. 10.7150/ijbs.6.51 20087442 PMC2808052

[B108] Villagra N. T. Navascues J. Casafont I. Val-Bernal J. F. Lafarga M. Berciano M. T. (2006). The PML-Nuclear Inclusion of Human Supraoptic Neurons: a New Compartment with SUMO-1- and Ubiquitin-Proteasome-Associated Domains. Neurobiol. Dis. 21, 181–193. 10.1016/j.nbd.2005.07.003 16125395

[B109] Walther T. C. Chung J. Farese R. V. Jr. (2017). Lipid Droplet Biogenesis. Annu. Rev. Cel Dev. Biol. 33, 491–510. 10.1146/annurev-cellbio-100616-060608 PMC6986389 28793795

[B110] Wang Y. MacDonald J. I. S. Kent C. (1995). Identification of the Nuclear Localization Signal of Rat Liver CTP:phosphocholine Cytidylyltransferase. J. Biol. Chem. 270, 354–360. 10.1074/jbc.270.1.354 7814396

[B111] Wang H. Gilham D. Lehner R. (2007). Proteomic and Lipid Characterization of Apolipoprotein B-free Luminal Lipid Droplets from Mouse Liver Microsomes. J. Biol. Chem. 282, 33218–33226. 10.1074/jbc.m706841200 17848546

[B112] Wang L. Wang Y. Liang Y. Li J. Liu Y. Zhang J. (2013). Specific Accumulation of Lipid Droplets in Hepatocyte Nuclei of PFOA-Exposed BALB/c Mice. Sci. Rep. 3, 2174. 10.1038/srep02174 23846197 PMC3709163

[B113] Wang H. Becuwe M. Housden B. E. Chitraju C. Porras A. J. Graham M. M. (2016). Seipin Is Required for Converting Nascent to Mature Lipid Droplets. Elife 5, e16582. 10.7554/eLife.16582 27564575 PMC5035145

[B114] Wang M. Wang L. Qian M. Tang X. Liu Z. Lai Y. (2020). PML2-mediated Thread-like Nuclear Bodies Mark Late Senescence in Hutchinson-Gilford Progeria Syndrome. Aging Cell 19, e13147. 10.1111/acel.13147 32351002 PMC7294779

[B115] Welte M. A. (2007). Proteins under New Management: Lipid Droplets Deliver. Trends Cel Biol. 17, 363–369. 10.1016/j.tcb.2007.06.004 17766117

[B116] Wilfling F. Wang H. Haas J. T. Krahmer N. Gould T. J. Uchida A. (2013). Triacylglycerol Synthesis Enzymes Mediate Lipid Droplet Growth by Relocalizing from the ER to Lipid Droplets. Develop. Cel 24, 384–399. 10.1016/j.devcel.2013.01.013 PMC3727400 23415954

[B117] Yan R. Qian H. Lukmantara I. Gao M. Du X. Yan N. (2018). Human SEIPIN Binds Anionic Phospholipids. Develop. Cel 47, 248–256. 10.1016/j.devcel.2018.09.010 30293840

[B118] Yoshizawa T. Yamagishi Y. Koseki N. Goto J. Yoshida H. Shibasaki F. (2000). Cell Cycle Arrest Enhances the *In Vitro* Cellular Toxicity of the Truncated Machado-Joseph Disease Gene Product with an Expanded Polyglutamine Stretch. Hum. Mol. Genet. 9, 69–78. 10.1093/hmg/9.1.69 10587580

[B119] Yue L. McPhee M. J. Gonzalez K. Charman M. Lee J. Thompson J. (2020). Differential Dephosphorylation of CTP:phosphocholine Cytidylyltransferase upon Translocation to Nuclear Membranes and Lipid Droplets. MBoC 31, 1047–1059. 10.1091/mbc.e20-01-0014 32186954 PMC7346725

[B120] Zhu J. Zhu S. Guzzo C. M. Ellis N. A. Sung K. S. Choi C. Y. (2008). Small Ubiquitin-Related Modifier (SUMO) Binding Determines Substrate Recognition and Paralog-Selective SUMO Modification. J. Biol. Chem. 283, 29405–29415. 10.1074/jbc.m803632200 18708356 PMC2570875

[B121] Zoumi A. Datta S. Liaw L.-H. L. Wu C. J. Manthripragada G. Osborne T. F. (2005). Spatial Distribution and Function of Sterol Regulatory Element-Binding Protein 1a and 2 Homo- and Heterodimers by *In Vivo* Two-Photon Imaging and Spectroscopy Fluorescence Resonance Energy Transfer. Mol. Cel Biol. 25, 2946–2956. 10.1128/mcb.25.8.2946-2956.2005 PMC1069603 15798184

